# Reply to TS Clark: ‘High-intensity-focused ultrasound in the treatment of primary prostate cancer: the first UK series’

**DOI:** 10.1038/sj.bjc.6605454

**Published:** 2009-12-08

**Authors:** H U Ahmed, M Emberton

**Affiliations:** 1 Division of Surgery and Interventional Sciences, University College London, London, UK; 2 NIHR University College Hospital/UCL Comprehensive Biomedical Research Centre, London, UK

**Sir,**

We are grateful to Clark for raising her concerns about our article on using high-intensity focused ultrasound (HIFU) to treat localised prostate cancer and the subsequent press interest within the UK (Ahmed *et al*, 2009). Outcomes are difficult to define in minimally invasive therapies of prostate cancer and there is no consensus about what is a successful outcome. What is certain is that prostate cancer is an over-diagnosed and over-treated disease for which treatments that can achieve low rates of incontinence and impotence are paramount. Through this independent, anonymous peer-reviewed paper, we have attempted to present our results in an open manner, with all data presented and summarised in such a way that has so far evaded this area of research. Incontinence is truly very low after HIFU. We thank Clarke for highlighting the weaknesses in the potency data. Our denominator, on which we have based the potency rates, is within the paper for all to evaluate. We have made no attempt to disguise this, although it is extremely difficult to fit all caveats and data into a short abstract. We are extremely grateful to the *British Journal of Cancer* for making available the full paper online for a period of time after publication without charge.

It is difficult to control press articles about research outcome. Reporters and editors of newspapers are interested in sound bites and headlines. The press release was written, edited and approved by Cancer Research UK and ourselves, as well as approved by the communication departments of a number of organisations involved in this piece of work. In addition, independent comments were made by the Prostate Cancer Charity and Cancer Research UK. They and ourselves were keen to emphasise the preliminary nature of these results and manage the expectations. In fact, all press coverage had quoted statements to this effect. Further, the NHS website covered the paper in detail and stated that ‘Headlines like “Prostate cancer treatment more successful than surgery” are not accurate at present’ (http//www.nhs.uk/news/2009/07july/pages/ultrasoundforprostatecancer.aspx).

However, we must not forget that everyday research is reported upon by press and media. This coverage is important as it generates interest, keeps the population informed, builds recruitment into prospective clinical trials that aim to answer the difficult questions of the day, and allows the public to gain an insight into the research work of the National Health Service and universities within the UK.
